# Conditional Learning Deficits in Children with ADHD can be Reduced Through Reward Optimization and Response-Specific Reinforcement

**DOI:** 10.1007/s10802-021-00781-5

**Published:** 2021-04-01

**Authors:** Hasse De Meyer, Gail Tripp, Tom Beckers, Saskia van der Oord

**Affiliations:** 1 Behaviour, Health and Psychopathology, KU Leuven, Tiensestraat 102, 3000 Leuven, Belgium; 2 Department of Psychology, Faculty of Behavioural Sciences, HELP University, Subang 2, Persiaran Cakerawala Seksyen, U4, 40150 Shah Alam, Selangor Malaysia; 3 Human Developmental Neurobiology Unit, Okinawa Institute of Science and Technology Graduate University, Okinawa 904-0495 1919-1 Tancha, Onna, Japan; 4 Leuven Brain Institute, KU Leuven, Leuven, Belgium; 5 Developmental Psychology, University of Amsterdam, Nieuwe Achtergracht 129, 1018 Amsterdam, Netherlands

**Keywords:** Attention Deficit Hyperactivity Disorder, Differential Outcomes, Conditional Discrimination Learning, Reward, Treatment

## Abstract

When children with ADHD are presented with behavioral choices, they struggle more than Typically Developing [TD] children to take into account contextual information necessary for making adaptive choices. The challenge presented by this type of behavioral decision making can be operationalized as a Conditional Discrimination Learning [CDL] task. We previously showed that CDL is impaired in children with ADHD. The present study explores whether this impairment can be remediated by increasing reward for correct responding or by reinforcing correct conditional choice behavior with situationally specific outcomes (Differential Outcomes). An arbitrary Delayed Matching-To-Sample [aDMTS] procedure was used, in which children had to learn to select the correct response given the sample stimulus presented (CDL). We compared children with ADHD (*N* = 45) and TD children (*N* = 49) on a baseline aDMTS task and sequentially adapted the aDMTS task so that correct choice behavior was rewarded with a more potent reinforcer (reward manipulation) or with sample-specific (and hence response-specific) reinforcers (Differential Outcomes manipulation). At baseline, children with ADHD performed significantly worse than TD children. Both manipulations (reward optimization and Differential Outcomes) improved performance in the ADHD group, resulting in a similar level of performance to the TD group. Increasing the reward value or the response-specificity of reinforcement enhances Conditional Discrimination Learning in children with ADHD. These behavioral techniques may be effective in promoting the learning of adaptive behavioral choices in children with ADHD.

## Background

ADHD is marked by elevated levels of inattention, hyperactivity and impulsiveness that are inconsistent with a child’s developmental stage (American Psychiatric Association, 2013). As a result of these symptoms, children with ADHD often fail to conform to the expected social, cognitive and emotional requirements of their environment, increasing their risk of adverse outcomes later in development (Wehmeier et al., 2010; Willcutt et al., 2005). A critical skill for adaptive socio-emotional and cognitive functioning is the ability to align one’s actions with the frequently changing expectations or requirements of the environment (e.g., being quiet and deferential in the classroom, being energetic and assertive in the playground) (Urcuioli, 2005). Numerous researchers have observed that children with ADHD have difficulty adapting their behavior to shifting environmental demands (Nigg & Casey, 2005; Sagvolden et al., 2005).

A widely used paradigm for testing behavioral adaptation to environmental expectations in laboratory research is a Conditional Discrimination Learning [CDL] task (Martínez et al., 2012; Mok et al., 2017). Conditional Discrimination Learning can be tested in an arbitrary Matching-To-Sample [aMTS] procedure in which associations have to be learned between non-similar, non-related sample stimuli and choice responses (Estévez et al., 2001; Trapold, 1970). On a given trial participants are presented with a single sample stimulus, e.g., S_1_, followed by a choice between two responses R_1_ and R_2_, and they have to learn to select the correct response. Importantly, which choice response is correct is dependent on the sample stimulus presented: R_1_ may be the correct choice for S_1_, but for another stimulus S_2_, R_2_ may be the correct choice response. When a retention interval is inserted between the offset of the sample stimulus and the appearance of the choice stimuli, the aMTS task becomes an arbitrary Delayed Matching-To-Sample [aDMTS] task (Case et al., 2015; Skinner, 1950). Due to its conditional nature, this instrumental learning task models the capacity to adapt choice behavior to situational requirements or hierarchical reinforcement contingencies (Mok et al., 2009).

Despite its clear clinical relevance, there is very little research on CDL in ADHD (for exceptions, see De Meyer et al., 2019; Gitten et al., 2006). The few available studies show no evidence for a deficit in CDL learning in children with ADHD compared to TD children when choice stimuli immediately follow the sample stimuli. However, in daily life a delay between environmental cues and behavioral choice is the rule rather than the exception (e.g., as when a child is instructed to begin a new task after first completing another one). We recently showed that under delay conditions (imposing a delay of 8 or 16 s between the sample stimulus and response choice), children with ADHD show poorer learning than TD children on a CDL task (De Meyer et al., 2019). In addition to being a good marker of children’s everyday ability to use environmental cues to adapt their behavior (Martínez et al., 2009), CDL delay tasks can potentially be used as an indicator whether intervention strategies, aimed at improving the ability to adapt behavior to contextual demands have the potential to work. This knowledge can be used for further development and testing of interventions in more ecologically valid designs and studies (e.g., micro trials; Staff et al., 2021).

One potential way to improve the degree to which children with ADHD adapt their behavior to environmental expectations, and thus to increase task performance on a CDL task, is by increasing the value of the associated reward. The use of a larger reinforcement (e.g., a large monetary reward, as compared to a small reward or feedback only) has been shown capable of normalizing the on-task performance of children with ADHD compared to TD children, including the amelioration of performance deficits linked to executive impairments (e.g., in working memory) in children with ADHD (Dovis et al., 2012; Fosco et al., 2015; Luman et al., 2005; Slusarek et al., 2001). Whether such an incentive-oriented manipulation would also be effective in improving CDL performance is to be determined.

Inspiration for a more cognitively oriented way to remediate impairments in adaptive choice behavior in children with ADHD can be found in the associative learning literature. Providing Differential Outcomes [DO] or response-specific reinforcement is a frequently used technique to overcome learning and memory deficits in clinical as well as non-clinical samples (Urcuioli, 2005). In DO, stimulus–response relationships are reinforced using response-unique, rather than general, outcomes; correctly choosing R_1_ after presentation of S_1_ will result in outcome O_1_, while correctly choosing R_2_ after presentation of S_2_ will result in a different outcome O_2_. This is in contrast to a non-Differential Outcomes [nDO] procedure, where different outcomes are provided randomly (O_1_ or O_2_), irrespective of the response, or the standard Common Outcomes [CO] procedure where only one outcome is used to signal correct responding across trials (Holden & Overmier, 2014; Overmier & Linwick, 2001). The use of a DO procedure allows for the formation of specific sample-outcome and response-outcome associations: besides an S-R association, an S-O association is formed that contains information on the specific outcome that can be earned for correct responding to the sample stimulus (Urcuioli, 2005). Given that this specific outcome is also linked uniquely to a specific choice response, the acquired S-O associations can help support correct choice behavior (i.e., the correct choice is not only supported by a direct S-R association but also by an indirect S-O-R associative chain) (Hochhalter & Joseph, 2001; Mok & Overmier, 2007). Thus, when the sample stimulus (S) is presented, it activates a prospective memory representation of a specific, to-be-earned outcome (S-O association) that primes a specific choice response. This prospective memory representation can help to overcome the memory load created by the imposition of a delay between the disappearance of the sample stimulus and the appearance of the choice stimuli (Overmier & Linwick, 2001).

In existing contingency management programs for children with ADHD, explicitly linking specific rewards to certain behaviors (e.g., differently colored stickers or marbles for various forms of situationally appropriate behaviors) as in a DO procedure, is to our knowledge not specifically being taught in behavioral management programs (Staff et al., 2021). There is substantial evidence that DO has beneficial effects on learning and memory in typically developing children and adults (Plaza et al., 2018; Urcuioli, 2005) and in other clinical groups (e.g., in Autism, Down’s, Prader-Willi and Korsakoff syndromes, and in Alzheimer’s disease) (Esteban et al., 2014; Hochhalter & Joseph, 2001; Joseph et al., 1997; Vivas et al., 2018). Studies in Korsakoff and Prader-Willi patients show benefits of DO in delayed and non-delayed conditional discrimination learning tasks (Hochhalter & Joseph, 2001; Joseph et al., 1997) and suggest that it has potential for targeting forgetfulness in daily life in clinical patients (e.g., for remembering the intake of medication). Given that Prader-Willi and Korsakoff syndromes share characteristics with ADHD (including the presence of impairments in learning and memory) (Hochhalter & Joseph, 2001), we speculate that the use of DO may facilitate learning and attenuate the performance deficit that children with ADHD exhibit in conditional discrimination learning under delays (De Meyer et al., 2019; Martínez et al., 2009, 2013; Overmier & Linwick, 2001).

Differential Outcomes procedures can be integrated in an aDMTS task in different ways, involving ﻿different types of outcomes. In a standard implementation of DO, the outcomes used can be conditioned (or secondary) reinforcers (e.g., a token) and/or primary reinforcers (Estévez et al., 2001; Martínez et al., 2009, 2013). The latter are hedonic reinforcers that are intrinsically motivating (e.g., food or water in animal research) (Estévez et al., 2001; Martínez et al., 2009, 2013). Whereas often in DO studies response-specific secondary and response-specific primary reinforcers are used as outcomes (e.g., response-specific tokens that can later be exchanged for token-specific candy), emerging evidence suggests that response-specificity at one level of outcome (e.g., only at the level of the secondary reinforcer) may be sufficient to achieve a beneficial DO effect. Then again, children with ADHD are known to process reinforcement differently than TD children and may need more optimal reinforcement than TD children to perform well on tasks (Dovis et al., 2012; Luman et al., 2010). When studying DO in children with ADHD, it is therefore important to determine whether response-specific primary and secondary reinforcement is superior to response-specific secondary reinforcement only.

In summary, the aim of the present study was to investigate whether enhancing the value of reward (remediation 1) and/or introducing differential outcomes (remediation 2) would ameliorate deficits in CDL performance, under conditions of delay, observed in children with ADHD compared to TD children. Within differential outcomes, we also assessed the importance of the degree of response-specificity of reinforcement; i.e., is there a difference in the effectiveness of response specific secondary reinforcer compared to response specific primary and secondary reinforcement.

**Fig. 1:**
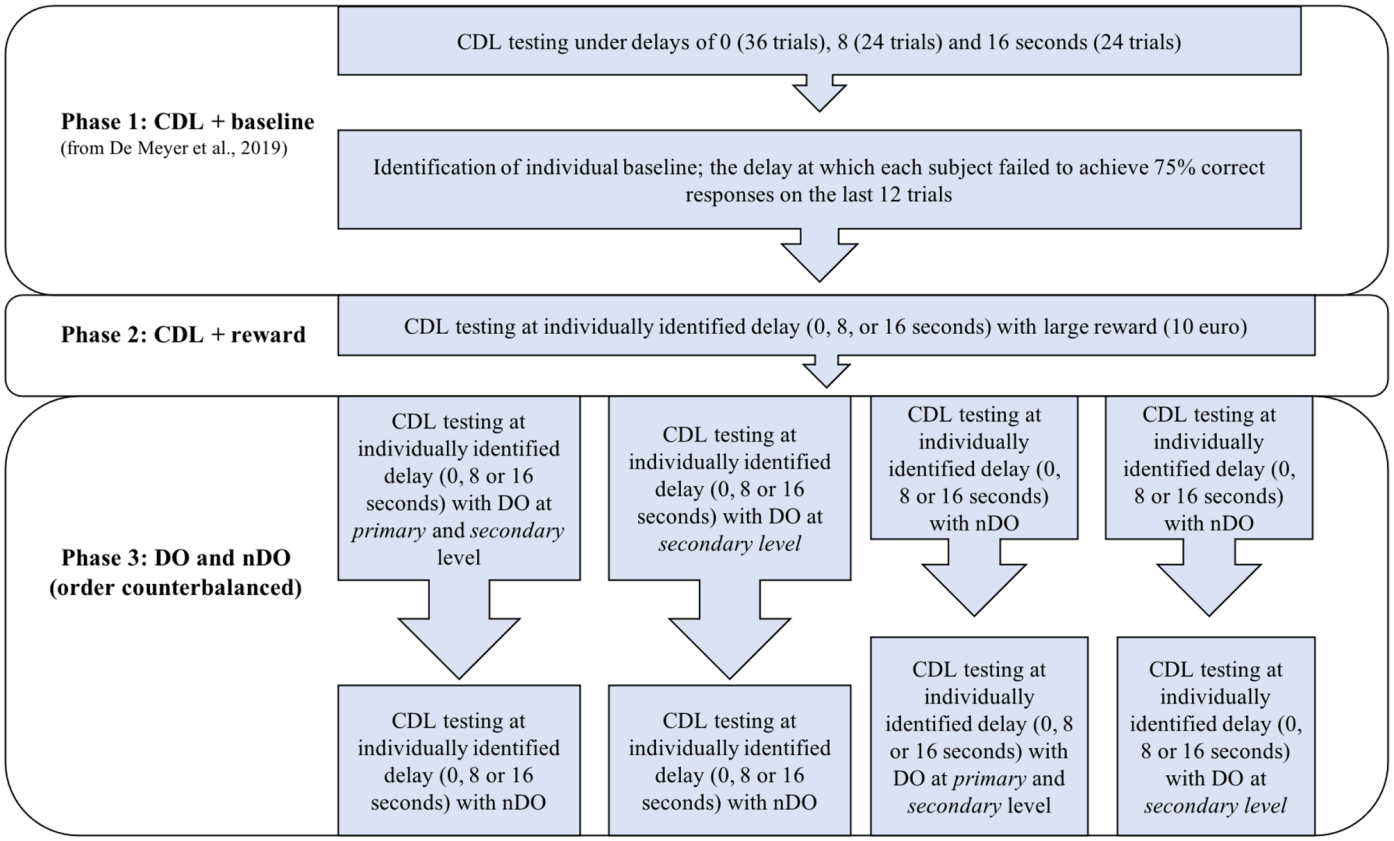
Study overview. Prior to the start of the current study, the delay at which CDL performance declined was determined individually through administration of aDMTS tasks with increasing delays between sample and choice stimuli (Baseline, phase 1). In phase 2, an aDMTS task with the same delay but including a monetary reward (possibility of obtaining 10 euros in addition to feedback only in the baseline condition) was administered. In phase 3, aDMTS tasks were administered under nDO and DO conditions (with order counterbalanced across participants). Within the DO manipulation, half of the participants were exposed to secondary reinforcement only and the other half to secondary as well as primary reinforcement, i.e., between-subject within the groups

Immediately before the start of the current study, all children had performed a series of CDL tasks with increasing delays to determine the delay at which their performance tapered off (De Meyer et al., 2019). It is that performance which serves as the baseline for the current study, and the associated delay which was used in the manipulations reported here (see Fig. 1 – Phase 1). Two different procedures were sequentially evaluated for their effect on the children’s CDL performance: (1) in the first CDL task, €10 could be accumulated through correct responding (large reward condition) (see Fig. 1 – Phase 2); (2) in subsequent CDL tasks, we used a within-subjects manipulation to compare the effects of DO and nDO on performance. In DO, making the correct choice led to unique outcomes and thus specific stimulus–response relations were learned. This differs from nDO where, non-specific outcomes were provided after correct choice responses, irrespective of the response (within-subjects) (see Fig. 1 – Phase 3). Based on previous research (Dovis et al., 2012; Luman et al., 2005; Slusarek et al., 2001), we expected that both procedures (the provision of larger reinforcers as well as the provision of DO reinforcers for correct choice behavior) would improve CDL performance under delay conditions, in the two groups, with a more pronounced effect for those with ADHD.

Between-subjects, we manipulated whether DO applied to secondary reinforcement only (with primary reinforcement being non-differential) or to secondary and primary reinforcement (see Fig. 1 – Phase 3). This was achieved by random assignment of the participants to either primary and secondary DO or secondary DO only. We predicted that ADHD and TD groups would both benefit more from a DO procedure where primary and secondary reinforcers were response-unique than from a DO procedure where only secondary reinforcers were response-unique. We expected these effects would again be more pronounced in children with ADHD, due to their altered reinforcement sensitivity.

## Method

Forty-six children with a prior diagnosis of ADHD (22 combined, 18 inattentive, and 6 hyperactive/impulsive) and 55 typically developing children participated in the study. Six participants (5 = TD, 1 = ADHD) were subsequently excluded due to an error in administration; i.e., an incorrect delay was selected from the baseline aDMTS tasks for use in the reward, nDO and DO tasks. The children, aged 8 to 12 years, were recruited through the clinical networks of the authors (ADHD group) and local schools (TD group). Study inclusion criteria were: (a) an estimated IQ score ≥ 80, based on the short form of the Dutch version of the Wechsler Intelligence Scale for Children [WISC-III-NL] (b) absence of any sensory, neurological or motor disorder or a clinical diagnosis of Autism Spectrum Disorder (as indicated by parents) (c) absence of a clinical diagnosis of Conduct Disorder [CD] as assessed by the CD section of the Disruptive Behavior Disorders module of the Diagnostic Interview Schedule for Children, Parent Version (PDISC; Shaffer et al., 2000) and (d) not taking any medication other than stimulant medication (in the case of ADHD) which participants were willing to withdraw 24 h prior to testing (Greenhill, 1998).

The diagnosis of ADHD was established by a certified psychiatrist or clinical psychologist and DSM-criteria were confirmed by the PDISC. Typically Developing children were required to fall within the normal range on the Inattentive and Hyperactivity/Impulsivity section (≤ 90.9^th^ percentile), Oppositional Defiant Disorder [ODD] section (≤ 95.2^nd^ percentile) and CD section (≤ 95.2^nd^ percentile) of the Disruptive Behavior Disorder Rating Scale (DBDRS; Dutch translation: Oosterlaan et al., 2008) as endorsed by parents.

### Measures

*WISC-III-NL, short version*: Vocabulary and Block Design, two subtests from the Dutch version of the WISC-III (Kort et al., 2005), were administered to estimate full-scale IQ. This composite score exhibits satisfactory validity and reliability (0.86 and 0.91) and is highly correlated with full-scale IQ (Sattler, 2001).

*PDISC:* The clinical assessment followed the algorithm of the Diagnostic Interview Schedule for Children, Parent Version (Shaffer et al., 2000). This interview, based on the DSM-IV criteria, has adequate psychometric properties (test–retest reliability = 0.79) and is a reliable assessment tool to assess DSM symptoms of ADHD, ODD and CD (Shaffer et al., 2000). The interviewers, licensed clinical psychologists or Masters students in clinical psychology, were trained by the first author in administering the PDISC.

*DBDRS*: The Dutch version of the Disruptive Behavior Disorder Rating Scale (Oosterlaan et al., 2008) contains four DSM-IV-TR based scales assessing Inattention, Hyperactivity/Impulsivity, ODD and CD symptoms. The 42-item questionnaire is designed to be completed by parents of children between six and sixteen years of age. Parents were asked to rate the behavior of their child on a 4-point Likert scale, ranging from 0 (*not at all*) to 3 (*very much*). Raw scores (ratings added across all symptoms) were transformed to norm scores ranging between 10 (50^th^ percentile, non-clinical) and 19 (99.9^th^ percentile, clinical). Adequate psychometric properties are reported for a Flemish sample; internal consistencies for the Inattention (α = 0.90), Hyperactivity/Impulsivity (α = 0.87), ODD (α = 0.88) and CD subscales (α = 0.66) (Oosterlaan et al., 2008) are moderate to high. ﻿

### Conditional Discrimination Learning Task: Baseline Assessment

**Fig. 2:**
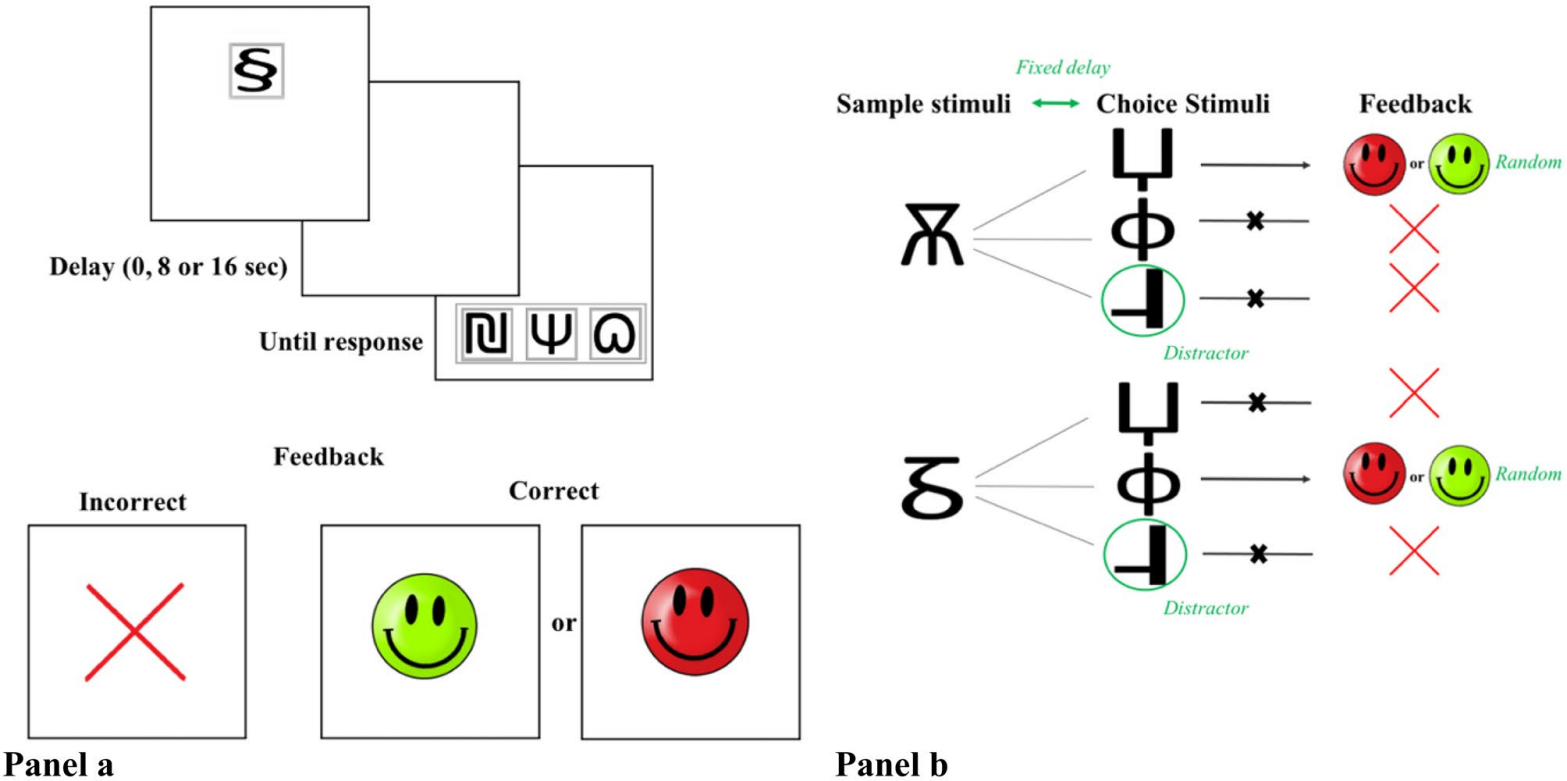
Panel a – Baseline aDMTS procedure (retrieved from De Meyer et al., 2019). A randomly chosen sample stimulus is presented at the top of a touchscreen. Upon touching the sample stimulus, the screen is cleared. After a delay of 0, 8 or 16 s, three choice stimuli appear and remain on the screen until the child responds by touching one of the stimuli. The child’s task is to learn to select the correct choice stimulus for a given sample stimulus through trial and error. Correct responding yields a green or red smiley (randomly determined), incorrect responding is followed by a red cross (see Fig. 1 – Baseline). After 2 s, the next trial is presented. Panel b – aDMTS task with increased reward. Children are presented with the aDMTS task with the relevant delay determined by their baseline performance. Correct responses yield smiley faces that accumulate towards a potential ten-euro reward accompanied by specific reinforcement instructions *“You have a higher chance at winning €10”.* Incorrect responses yield a red cross (see Fig. 1 – Phase 2)

In phase 1, initial CDL performance was assessed through repeated arbitrary Delayed Matching-To-Sample [aDMTS] tasks, as reported in De Meyer et al. (2019).^1^ In each aDMTS task, participants learnt arbitrary relationships between a new set of sample stimuli and choice stimuli (see Fig. 2 – Panel a); conditional upon the presentation of sample stimulus S_1_, selection of C_1_ is the correct response and upon presentation of S_2_, selection of C_2_ is the correct response. Children learnt the correct (S_1_-C_1_ and S_2_-C_2_) associations through a feedback-based trial-and-error procedure; correct responses were followed by a smiley face, incorrect responses were followed by a red cross. To increase the level of difficulty, a third choice stimulus C_3_ was added on all trials. In order for participants to become acquainted with the task, a training phase was presented prior to the first CDL task.

Across the task, there was a gradual increase in the retention interval between sample and choice stimuli (from 0 s through 8 s to 16 s) (see Fig. 1 – Baseline). Each retention interval involved 24 trials (with the exception of the 0-s task, which included 36 trials). The appearance of the sample stimulus (S_1_ or S_2_) and the position of the choice stimuli (C_1_, C_2_, C_3_) was determined randomly for each trial (12 options) and a different set of stimuli was used for each delay. The correct sample-choice association was determined in advance and not counterbalanced. With increasing delay between the sample and choice stimuli, a drop in learning performance is consistently observed, typically attributed to an increased memory load (Case et al., 2015). The sample and choice stimuli for each CDL task were clearly distinguishable, randomly chosen abstract figures from MS Word 2008 presented in black on a white background square measuring 5 × 5 cm. Outcome stimuli were colored 10 × 10 cm smiley figures. The task was presented on a 15-inch touchscreen.

After performing the basic aDMTS tasks with increasing delays, the delay at which a participant failed to reach the criterion of 75% correct choices over the last 12 trials was used as the delay for that participant in the current study; final performance at that delay during the basic aDMTS tasks is used here as the children’s *baseline*. If participants achieved criterion for all tested delays, the 16-s delay was used for the current study and performance on that delay was used as the baseline to which both of the remediation procedures were compared.

### Conditional Discrimination Learning Task: Reinforcement Manipulations

For the first reinforcement manipulation (phase 2), (see Fig. 2 – Panel b), the aDMTS task, with an individually determined delay (see figure legend) was presented that included a 10 euro monetary reward for correct choice behavior. At the beginning of the task, participants were informed about the change in reward outcome: ‘*From now on, for every smiley you will earn a point. The more points you earn, the higher the chance you have at winning ten euros. When you have earned enough points, the game will end and you will see a green screen’*, which was assumed to maintain motivation over time (Dovis et al., 2012). ﻿The ten one-euro coins that the child could earn were shown and placed in sight but out of reach; they remained in view throughout the entire task. All children received 10 euros at the end of testing, irrespective of their actual performance on the task.

**Fig. 3:**
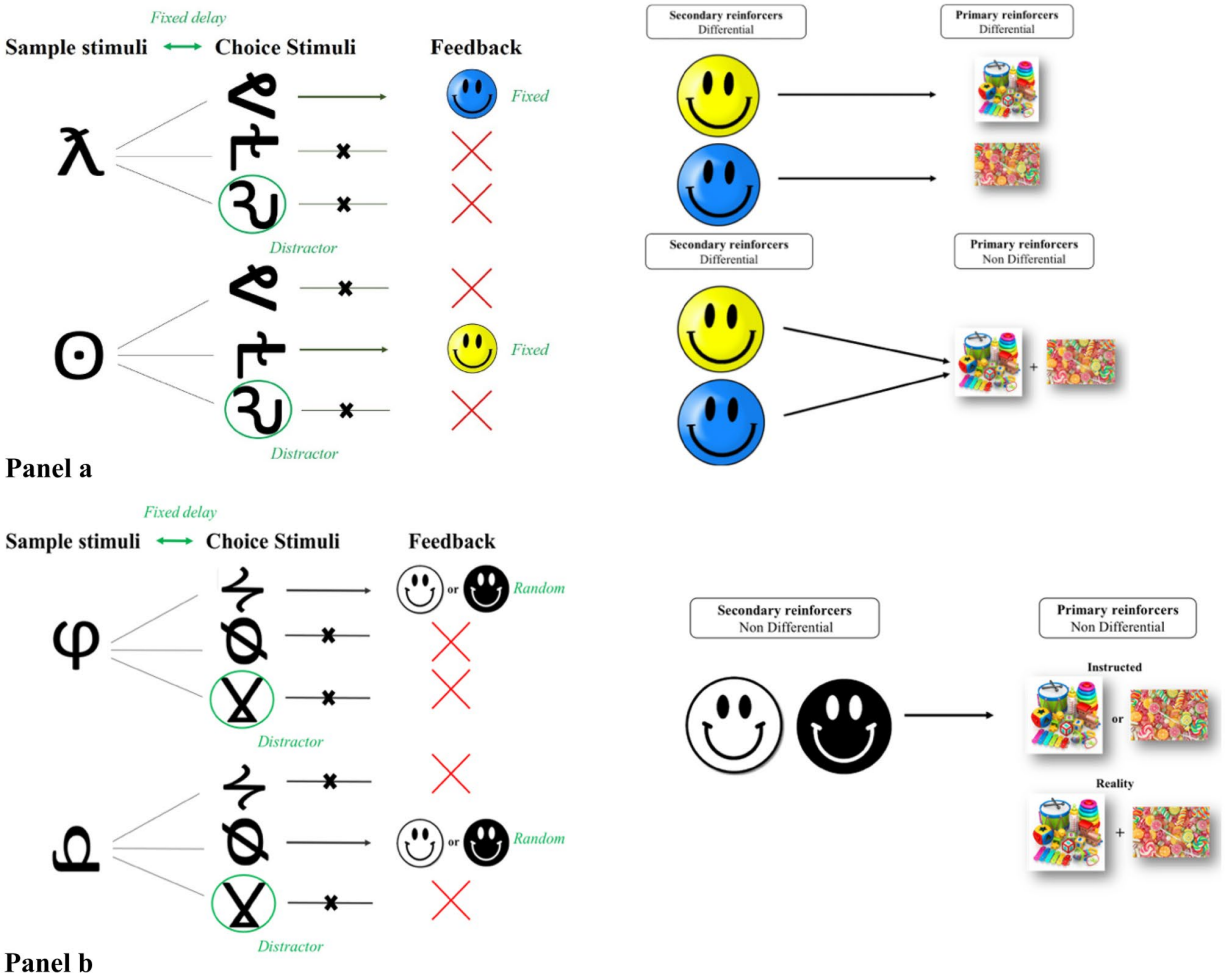
Panel a – DO aDMTS task. The basic aDMTS task was modified so that correct responses yield a sample-specific outcome (DO). All participants receive differential secondary reinforcers (smileys of a sample-specific color). For half of the participants, those secondary reinforcers accumulate towards differential primary reinforcers (smileys of one color earn a toy reward, the other earns candy), for the other participants, secondary reinforcers accumulate towards non-differential primary reinforcers (both types of smileys earn candy and toy rewards) (see Fig. 1 – Phase 3). Panel b – nDO aDMTS task. An aDMTS is presented in which correct responses yield a non-specific outcome (nDO): Participants always receive non-differential secondary reinforcers (randomly white or black smileys) that accumulate towards a non-differential primary reinforcer (toys or candy, as instructed and determined by the experimenter) (see Fig. 1 – Phase 3). In reality, children were rewarded with both toys and candy (explained as being a consequence of their outstanding performance) unrelated to their actual performance to control for reward level across conditions and the potential influence on their motivation

In phase 3, we evaluated the second reinforcement manipulation, that is if CDL performance could be improved through the use of Differential Outcomes as compared to non-Differential Outcomes. The order of nDO/DO and nature of DO (primary and secondary vs secondary) were varied between participants, stratified for gender, age, and group. All children performed both the nDO and DO tasks (within-subjects). The nature of the DO task (primary and secondary DO or secondary DO only) was manipulated between subjects. In the DO aDMTS task, correctly choosing C_1_ after the presentation of S_1_ resulted in outcome O_1_ (a blue smiley), whereas correctly choosing C_2_ after presentation of S_2_ resulted in a different outcome O_2_ (a yellow smiley) (see Fig. 3 – Panel a). In order to test the influence of the degree of response-specificity of reinforcement (i.e., response specificity of secondary reinforcement only versus primary and secondary reinforcement), half of the children in each group (ADHD, TD) received primary and secondary DO, in that they were told that different smileys could later be exchanged for different rewards: O_1_ (blue smiley) accumulated towards candy and O_2_ (yellow smiley) towards a toy. At the beginning of the task, participants were informed about the change in reward outcome: *For every yellow smiley you will earn yellow points and for every blue smiley you will earn blue points. At the end, you can exchange all the yellow points for toys and all the blue points for candy. The more yellow points you earn, the higher the chance you have at earning toys. The more blue points you earn, the higher the chance you have at earning candy.* The other half of the children in each group received secondary DO only. They were told that secondary reinforcers accumulate towards non-differential primary reinforcers (both types of smileys earn candy and toy rewards); *For every blue smiley you will earn blue points and for every yellow smiley you will earn yellow points. At the end, you can exchange all the blue points and all the yellow points together for toys and candy. The more points you earn, the higher the chance you have at earning candy and toys* (see Fig. 3 – Panel a). All task instructions were explained to the participants and the researcher checked whether they understood all instructions. Participants were told that they needed to obtain enough smileys in order to receive a reward. In effect, all children received identical rewards after the programmed 24 trials irrespective of their performance. For the task, the sample stimuli, choice stimuli and correct association were randomly determined by the computer program.

In the nDO task, which could be presented before or after the DO task, correct responses yielded a randomly colored smiley, i.e., either a black or white smiley, unrelated to the sample presented (see Fig. 3 – Panel b). Children were told that smileys accumulated towards a reward (toys or candy, determined by the experimenter), in a non-differential way; *For every smiley you will earn a point. At the end, you can exchange all the points for candy or toys. The more points you earn, the higher the chance you have at candy or toys.* To control for reward level across conditions (i.e., DO and nDO) and the potential influence on their motivation, upon completing the 24 trials in the nDO task, children were rewarded with both toys and candy (explained as being a consequence of their outstanding performance) but unrelated to their actual performance (see Fig. 3 – Panel b).

In all tasks, CDL performance was measured as *the percentage of correct responses* across all trials of the task minus the first four (performance on the first four trials is determined by chance only). For each participant, this metric was calculated for 1) the *baseline* task (i.e., the aDMTS task from the baseline phase on which a participant failed to reach the 75% criterion, with this delay used in all subsequent tasks; 2) the aDMTS task from Phase 2 (increased reward); and 3) the two aDMTS tasks from Phase 3 (nDO and DO)*.*

## Procedure

Before participating in the study, separate information letters were given to parents and children, and informed consent was obtained from both parents. While parents completed the structured interview (ADHD group only) and questionnaires (DBDRS and demographic questionnaire), children performed the experimental tasks in a distraction-free room. All participants completed the basic aDMTS task immediately before the tasks described here (*baseline*; for full results, see De Meyer et al., 2019). After determining each participant’s baseline delay, used in all subsequent tasks, the aDMTS task with monetary reward was conducted (Phase 2). All children received the monetary reward immediately afterwards. Next, children performed the nDO and DO aDMTS tasks, in counterbalanced order (Phase 3). All tasks were separated by a 10-min break. The experimenter remained in the room throughout the testing procedure (± 100 min).^2^ All children were able to complete the tasks and families were compensated with an additional 10 euros for participating in the study.

The study was approved by the KU Leuven Social and Societal Ethics Committee (G-2015 01 156). The authors confirm that the study was conducted in line with the ethical standards of the institutional research committee and with the 1975 declaration of Helsinki and its 2008 amendment.

## Results

Review of the distribution of the outcome variables detected some extreme values (outliers) and high skewness and kurtosis for all outcome variables (unrelated to group), indicating non-normal distributions. Therefore, data was subjected to an arcsine transformation, as is recommended when outcome variables are percentages (see also IBM Corp. 2019; Zar, 1984).^3^ After transformation, one extreme outlier^4^ was detected using boxplots and deleted from the dataset. For one other participant the last 4 of 24 trials in the DO condition were missing. These missing values were replaced by the average score of the group (TD) for this variable (Field, 2013). The ADHD and TD groups did not differ in mean age or family education level but did differ in gender distribution (*χ*^*2* =^ 4.62, *p* = 0.032) with an uneven distribution of boys (N = 31) and girls (N = 14) in the ADHD group compared with the control group (see Table 1 and the appendix for demographic characteristics). As often observed in studies of children with ADHD, there was a main effect of group for IQ with children in the TD group scoring higher than children with ADHD, *F*(1, 92) = 10.89, *p* = 0.001, *ηp*^*2*^ = 0.106. Neither IQ nor gender were included as covariates in the analysis as neither variable correlated with any of the outcome variables.

**Table 1:** Demographic and Clinical Characteristics for the ADHD and TD children

	ADHD		TD			
	M(SD)		M(SD)		*F/χ*^*2*^	*p*
*Gender N*					4.62	0.032*
Male (N/%)	31 (68.89)		23 (46.94)			
Female (N/%)	14 (31.11)		26 (53.06)			
Age (years)	10.29 (0.99)		10.07 (1.21)		0.90	0.346
FSIQ	98.00 (11.72)		105.35 (9.84)		10.89	0.001**
Dyscalculia (N/%)	1 (2.22)		0			
Dyslexia (N/%)	4 (8.88)		0			
ODD (PDISC – N/%)	44 (46.80)		-			
Medication (N/%)	23 (51.11)		0			
Maternal education^1^	1.93 (0.82)		1.72 (0.74)		2.04	0.360
*DBDRS (norm scores)*^2^						
Inattention	15.02 (2.01)		10.53 (1.00)		191.56	< 0.001***
Hyperactivity/Impulsivity	14.23 (2.44)		10.51 (0.98)		96.51	< 0.001***
ODD	12.42 (2.43)		10.71 (1.24)		18.57	< 0.001***
CD	11.42 (1.53)		10.96 (1.15)		2.68	0.105
*PDISC (number of symptoms)*						
Inattention	7.43 (1.65)		-			
Hyperactivity/Impulsivity	6.00 (2.57)		-			
ODD	3.23 (2.14)		-			
CD	0.45 (0.79)		-			

*ADHD *Attention Deficit Hyperactivity Disorder, *TD* Typically Developing, *FSIQ *Full Scale IQ, *PDISC *Diagnostic Interview Schedule for Children, Parent Version, *DBDRS *Disruptive Behavior Rating Scale *ODD *Oppositional Defiant Disorder, *CD* Conduct Disorder

*p < .05, **p < .01, ***p < .001

^a^High (1) = University Education; Average (2) = Non-University Higher Education; Low (3) = Secondary Education (or less); 3 missing data points (1 = ADHD; 2 = TD)

^b^2 missing data points for ADHD group

To determine if adding a large reward or changing the associative structure of the task improved CDL performance, two group x task repeated-measures ANOVAs were run, the first comparing performance on the baseline aDMTS task with the monetary reward aDMTS task across the ADHD and TD groups. The second one comparing performance on the DO and nDO aDMTS tasks across the two groups. Additionally, a group x condition fixed-factors ANOVA was conducted to compare CDL performance between the two groups (ADHD/TD) when the DO manipulation involved secondary reinforcement only versus primary and secondary reinforcement. Post-hoc analyses, independent-samples *t*-tests and paired-samples *t*-tests were conducted to identify the source of the significant interaction effects. Effect sizes are reported for ease of interpretation; small (*ηp*^*2*^ = 0.01; *d* = 0.2); moderate (*ηp*^*2*^ = 0.06; *d* = 0.5) and large (*ηp*^*2*^ = 0.14; *d* = 0.8) (Cohen, 1988, 1992).

The distribution of the individually determined delays, selected on the basis of performance in the baseline tasks, was not significantly different between the groups, *χ*^2^ (2) = 5.75, *p* = 0.056. For the majority of children with ADHD and all TD children, a delay of 16 s was selected (ADHD: *n* = 40, TD: *n* = 49); for the remaining children in the ADHD group, an 8-s (*n* = 3) or 0-s (*n* = 2) delay was selected.

**Fig. 4:**
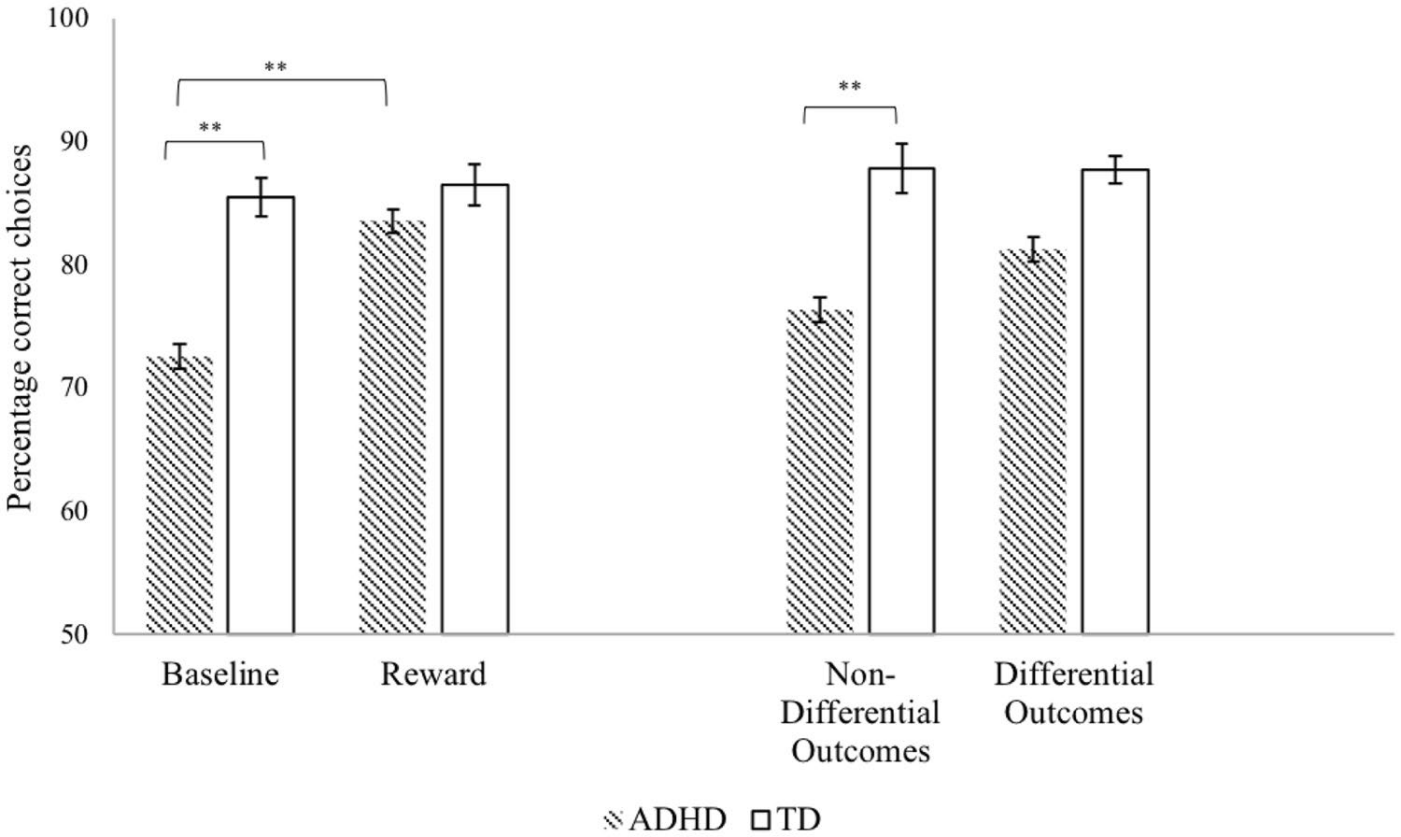
CDL performance across tasks for children with ADHD and TD children. For ease of interpretation untransformed data is displayed. **p < 0.01

The first 2 (group: ADHD vs TD) × 2 (condition: baseline vs monetary reward) repeated measures ANOVA yielded statistically significant main effects for condition, *F*(1, 92) = 6.97, *p* = 0.010, *ηp*^*2*^ = 0.070 and group, *F*(1, 92) = 9.45, *p* = 0.003, *ηp*^*2*^ = 0.093, and a significant condition by group interaction,^5^*F*(1, 92) = 4.60, *p* = 0.035, *ηp*^*2*^ = 0.048; adding a monetary reward had a larger impact on the CDL performance of children with ADHD compared to TD children (see Fig. 4, Table 2). Follow-up independent-samples *t*-tests indicated that children with ADHD differed significantly from the TD children at baseline *t*(79.18)^6^ = 3.29, *p* = 0.002, *d* = 0.69, but not following the addition of a monetary reward, *t*(92) = 1.56, *p* = 0.122, *d* = 0.32. Paired-samples *t*-tests showed that the difference in CDL performance between the baseline task and the monetary reward task performance was significant for children with ADHD, *t*(44) = -2.85, *p* = 0.007, *d* = 0.43 but not for TD children, *t*(48) = -0.44, *p* = 0.666, *d* = 0.06.

**Table 2:** Percentage of Correct Responses (with Standard Deviations)^1^ and Univariate ANOVA Results^2^ for Baseline, Reward, Differential Outcomes and Non-Differential Outcomes Tasks in the ADHD and TD groups

	ADHD		TD				
	M (SD)		M (SD)		*F*	*p*	*ηp*^*2*^
Baseline	72.53 (19.93)		85.51 (11.10)		11.10	0.001**	0.108
Reward	83.56 (12.23)		86.53 (11.69)		2.43	0.122	0.026
*Differential Outcomes*	81.22 (16.89)		88.01 (7.72)		3.71	0.057	0.039
DO—secondary	80.42 (19.39)		88.94 (7.81)		2.15	0.149	0.043
DO—primary & secondary	82.14 (13.93)		86.96 (7.65)		1.53	0.223	0.035
Non-Differential Outcomes	76.33 (21.52)		87.86 (14.22)		12.71	0.001**	0.121

ADHD Attention Deficit Hyperactivity Disorder; TD Typically Developing

^**^p < .01

^1^Based on untransformed data

^2^Based on transformed data

The effect of DO versus nDO was compared between groups in a 2 (condition; DO vs nDo) × 2 (group; ADHD vs TD) repeated measures ANOVA.^7^ There was a significant main effect of group, *F*(1, 92) = 10.87, *p* = 0.001, *ηp*^*2*^ = 0.106, but not condition, *F*(1, 92) = 0.19, *p* = 0.665, *ηp*^*2*^ = 0.002. The group x condition interaction was significant, *F*(1, 92) = 5.37, *p* = 0.023, *ηp*^*2*^ = 0.055 (see Fig. 4, Table 2). Follow-up paired-samples and independent-samples *t*-tests showed that performance did not differ significantly between nDO and DO for either group, *t*(48) = -1.46, *p* = 0.152, *d* = 0.23 (TD) and *t*(44) = 1.79, *p* = 0.081, *d* = 0.26 (ADHD), however for children with ADHD, compared to TD children, the percentage of correct choices was significantly lower under nDO, *t*(92) = 3.57, *p* = 0.001, *d* = 0.74, but not under DO, *t*(70.88)^8^ = 1.89, *p* = 0.063, *d* = 0.39. The order in which the conditions were presented did not influence the results when included as a covariate.

**Fig. 5:**
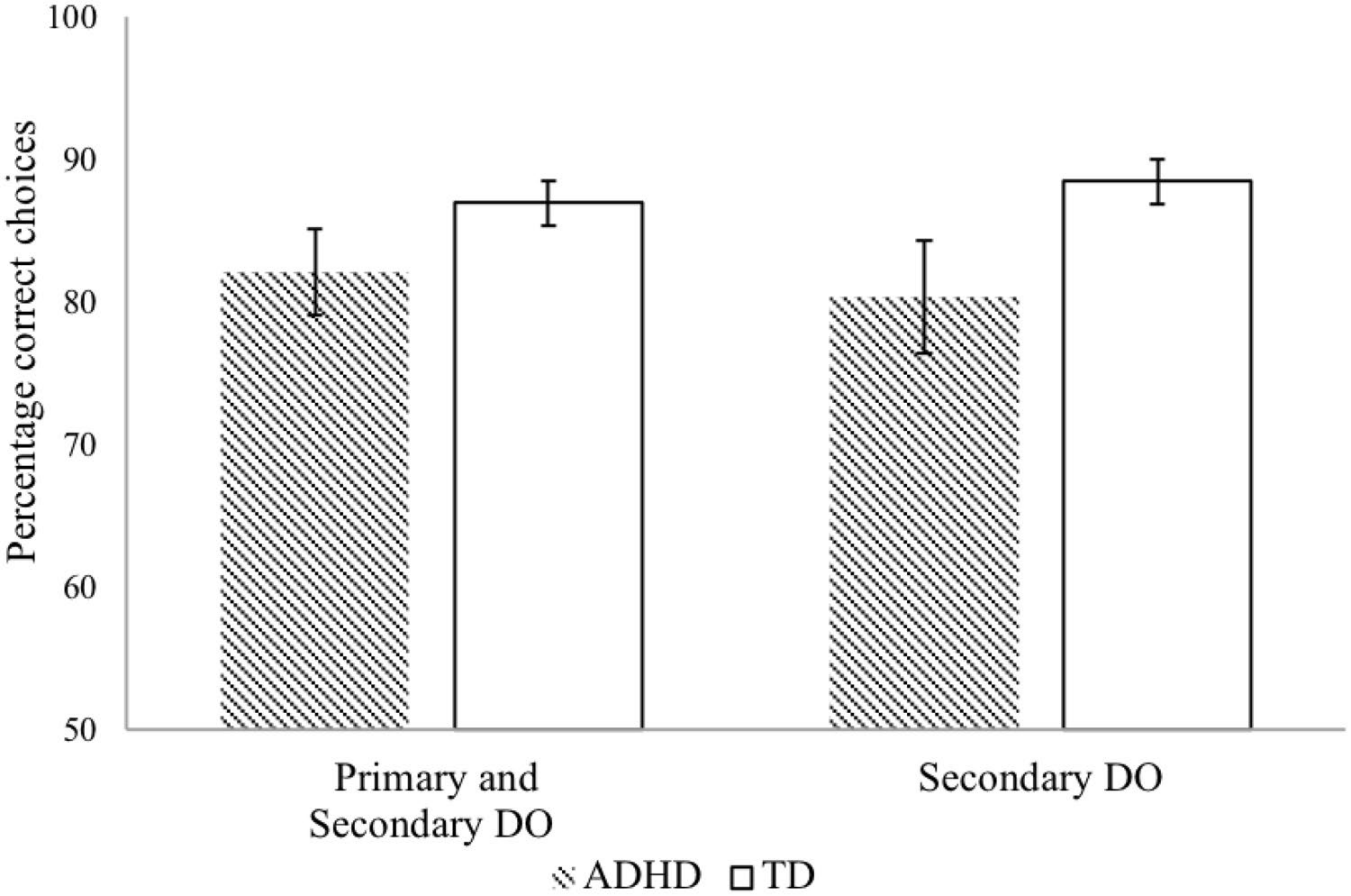
DO performance across conditions for children with ADHD and TD children. For ease of interpretation untransformed data is displayed

To explore whether a combination of response-specific secondary and primary reinforcement is more effective on CDL performance than response-specific secondary reinforcement only, a 2 (condition; secondary vs primary and secondary) × 2 (group; ADHD vs TD) factorial ANOVA was run. Results showed that there was no significant group, *F*(1, 90) = 3.56, *p* = 0.062, *ηp*^*2*^ = 0.038, or condition effect, *F*(1, 90) = 0.23, *p* = 0.636, *ηp*^*2*^ = 0.003, nor a group by condition interaction *F*(1, 90) = 0.11, *p* = 0.740, *ηp*^*2*^ = 0.001 (see Fig. 5).^9^ Applying DO at secondary level only or at both primary and secondary level did not differentially impact performance in the two groups.

## Discussion

Children with ADHD experience difficulty in using feedback to adapt their behavior in the presence of delays (Conditional Discrimination Learning). Here we evaluated the ability of different reinforcement manipulations to improve performance on a CDL task. Specifically, we tested the effects of increasing reinforcer size and the introduction of Differential Outcomes (i.e. response-specific reward outcomes). Within DO, we explored whether response-specific primary and secondary reinforcement was superior to response-specific secondary reinforcement only.

Contrary to our prediction that increasing reward size or value would have a positive effect on CDL performance under conditions of delay in both groups, we found a significant improvement in performance, i.e., a higher percentage of correct responses compared with baseline conditions, in the ADHD group only. Under increased reward conditions, the performance of the ADHD and TD group was no longer significantly different, suggesting a normalization of performance for the ADHD children.

Similarly, adding a specific reward outcome to sample-choice associations, i.e., DO, improved performance on the delayed CDL task in children with ADHD only. Although a significant difference in performance was found between the groups when using non-differential outcomes, under DO reward conditions the difference between the ADHD group and the TD group was no longer significant. Again, this suggests that adding DO may normalize CDL performance in children with ADHD. Further, we predicted that manipulating the nature of the reward within DO would affect performance. The data do not support this hypothesis, i.e., primary and secondary DO performance did not differ from performance under secondary DO only, for either group.

The observation of improved CDL performance through reward maximalization is in accordance with earlier findings that indicate beneficial effects of reward optimization on deficits in Executive Functioning [EF] performance in ADHD (Dovis et al., 2012; Fosco et al., 2015; Slusarek et al., 2001). In a DMTS task, a drop in accuracy under conditions of delay is often attributed to deficits in short-term (Etkin & D’Amato, 1969; Roberts & Grant, 1978) or working memory (Case et al., 2015; Kempton et al., 1999), although the literature has not addressed which specific memory aspect is involved in aDMTS. Our previous study (De Meyer et al., 2019), however, suggested that neither short-term nor working memory was related to performance on the aDMTS CDL task. The task used to evaluate memory in that study (Corsi Block Tapping Task; visual-spatial memory), may not have assessed memory components required for CDL learning. The current study shows that adding a reward improves performance on a CDL task under delay, although the specific mechanisms responsible for this improvement have yet to be determined.

Improvement of CDL performance through a monetary reward involves an increase in reward value, which might have served to remediate an underlying memory (short-term or working) deficit. The impact of DO on CDL performance might likewise be mediated by an effect on memory; the nDO and DO conditions did not differ in reward *size*, but rather in the *specificity* of reward. Although the increase in performance from nDO to DO in children with ADHD failed to reach significance (*p* = 0.081), DO did appear to “normalize” performance in the ADHD group, i.e., they performed more similarly to TD children on CDL. One explanation for the effect of a DO procedure on aDMTS performance is that it allows for the associative activation of a prospective memory representation upon presentation of the sample stimulus that primes correct choice behavior. This extra memory representation might have helped to counter possible short-term or working memory deficits that could impair aDMTS CDL performance in children with ADHD.

An alternative explanation for increased performance on the CDL task through increased reward or DO centers on their emotional-motivational effects (Sonuga-Barke, 2002). Within a CDL task, the delay between the sample and the choice stimuli (and the associated reward) can trigger a negative emotional state in children with ADHD, known as Delay Aversion (Antrop et al., 2006), which can result in inattentive and hyperactive symptoms and lead to decreased task performance (Marco et al., 2009). The addition of a more salient reinforcer or DO possibly helps to overcome such aversion caused by delay, e.g., through increased attention towards the sample-choice association. However, our previous study did not find an association between delay aversion and CDL performance under a delay (De Meyer et al., 2019), rendering this explanation less likely. Alternatively, a more rewarding or response-unique outcome may simply serve to increase the motivation of children with ADHD to perform the CDL task as well as possible. However, given reward intensity was equal across the DO and nDO conditions, a simple motivational account does not offer a convincing explanation for the effects of DO. Nevertheless, the results do indicate an improvement in CDL performance through use of a large monetary reward for children with ADHD, removing the significant difference in performance between ADHD and TD groups that was observed at baseline.

Contrary to the results of earlier studies (e.g., Martínez et al., 2013; Mok & Overmier, 2007; Molina et al., 2015) and our own predictions, associating a specific outcome to a stimulus-choice association (DO) did not significantly improve the performance of TD children. This may be due to a ceiling effect in the baseline performance of TD children, leaving limited room for change. Findings from previous research suggest a facilitating effect of DO on performance only when the task is sufficiently challenging (e.g., a 4-cue task for adults) (Estévez et al., 2001; Maki et al., 1995; Miller et al., 2002). Despite care in task development (balancing task difficulty for both groups through pilot studies), DO would perhaps only facilitate performance in TD children in a more challenging task design.

Additionally, it was hypothesized that the use of response-specific secondary and primary reinforcement would enhance performance as compared to response-specific secondary reinforcement only. Unexpectedly, performance was similar in both DO conditions. It may be that presenting a response-specific secondary reinforcer is already potent enough to create a performance ceiling effect, thereby leaving little room for further improvement with response-specific primary reinforcement. It is also possible that the absence of a difference between these two forms of DO is related to the similarity in instructions between primary DO only and primary and secondary DO. The instructions given to the children in both DO conditions were quite similar (see Appendix), with children being promised candy and a toy in each (be it differential in one condition but not the other).

The current study comes with some caveats. To begin with, we did not succeed in collecting teacher ratings to confirm the ADHD diagnosis for all participants due to practical constraints (i.e., no response, children changing teachers, absence of contact information) and therefore cannot confirm the cross situational severity of symptoms, considered a core diagnostic criterion in the diagnosis of ADHD. While acknowledging this limitation, most of the children who entered the study had been previously assessed and diagnosed through the KU Leuven university hospital, by means of multi-method, multi-informant assessments where also cross-situational severity was taken into account. Over the course of the study (including the baseline testing reported in De Meyer et al., 2019), the aDMTS task was administered four times. An influence of repeated task administration on performance cannot be excluded, although the significant group difference in nDO performance (involving either the third or fourth aDMTS task, depending on counterbalancing) and the absence of a significant within-group difference between the baseline and nDO performance, *t*(93) = -1.74, *p* = 0.086, argue against a simple task training effect. The current design did not allow us to control for the contextual effect of being rewarded with a monetary reward before the DO-nDO conditions, as the monetary reward condition was always presented first, after which DO and nDO were presented in counterbalanced order. Therefore, we cannot strictly rule out that delivering a large monetary reward ahead of the DO/nDO tasks differentially affected performance of the ADHD and TD groups. Another possible limitation relates to the stimuli used in the aDMTS task. Over the four aDMTS tasks (baseline, reward, DO, nDO), the set of stimuli used was fixed and not counterbalanced. Therefore, performance differences between aDMTS manipulations might, in principle, be due to stimuli-specific characteristics. However, considerable care was taken to establish stimulus sets of equal difficulty. In addition, it is possible that the task instructions influenced reward expectations differently for the DO and nDO conditions, favoring the DO condition. Although this effect was not evident in the performance of TD children (equal performance in DO vs nDO) we cannot rule out an ADHD specific differential effect. Finally, due the between-subjects manipulation of DO (primary and secondary versus secondary DO only), groups were rather small, reducing power to detect significant differences between those two forms of DO.

Despite the promising results, questions remain regarding the mechanism underlying the DO phenomenon effect. Further work is needed to disentangle what underlies the effectiveness of DO. This is important to provide a better understanding of its positive effects for children with ADHD. In future studies it would be important to test whether a DO effect can also be achieved through other types of response-specific reinforcers (e.g., differential versus non-differential social reinforcers).

## Clinical Implications

The findings of this study have a number of implications for maximizing the impact of operant techniques in behavioral treatment for ADHD. In Behavioral Parent Training [BPT], a token economy is a widely used operant technique with the core aim of increasing adaptive and reducing inappropriate behavior in children with ADHD (Sullivan & O’Leary, 1990). In a token economy, children are rewarded with specific tokens (e.g., marbles, stickers, etc.) for adaptive behavior. A standard token economy, however, applies only one token type to target various forms of situationally appropriate behaviors (e.g., a sticker for sitting still during mathematics *and* for playing nicely with siblings) (Coelho et al., 2015). Our results suggest that applying response-specific reinforcers may increase the learning of situation-specific stimulus–response associations in children with ADHD. This differential rewarding approach has already proven beneficial in a range of clinical populations (Esteban et al., 2014; Hochhalter & Joseph, 2001; Overmier & Linwick, 2001) and is often implemented in token economy programs for children with Autism Spectrum Disorder (Fairbanks & Sugai, 2014; Neitzel, 2010), a neurodevelopmental disorder with a significant overlap in clinical behavioral features and etiology with ADHD (Craig et al., 2015).

While the increase of reward value through monetary means also increases performance in children with ADHD, realistically, the addition of a high-value reward (e.g., 10 euro) is less feasible in real-life situations compared to the relatively simple implementation of DO. In the present study, DO was as effective as a high value of reward in increasing CDL performance in children with ADHD, and the implementation of DO in token economy programs can be relatively easy (e.g., rewarding on-task behavior with a red token and rewarding the raising of a hand before answering with a blue token).

In conclusion, the present research provides initial evidence that deficits in delayed conditional discrimination learning in ADHD on a DMTS task can be attenuated by enhanced reward and DO manipulations. Our results have potential implications for the refinement of behavioral interventions for children with ADHD. These findings can, for example inspire adaptations to existing token economies in ADHD, and further testing of these adapted “differential outcomes” token economies versus “non-differential” token economies on proximal daily life outcomes in micro-trails (Staff et al., 2021). Further research should also be directed at a better understanding of the mechanisms through which increased reward and DO exert their beneficial effects on CDL performance.

## Supplementary Information

Below is the link to the electronic supplementary material.

Supplementary file1 (DOCX 19 KB)

## Footnotes

1 A detailed description of the aDMTS task is provided in De Meyer et al., 2019.

2 The tasks were part of a larger test battery including tasks on working memory and delay aversion (see De Meyer et al., 2019).

3 Data analysis on transformed and untransformed data was applied (both parametric and non-parametric), leading to similar results.

4 Performance that is more extreme than three times the length of the boxplot (between the 25^th^ and 75^th^ percentile), as marked with an asterisk (*) on the boxplot.

5 We analyzed the data including only those children tested with a 16 s-delay. The main effects of condition and group remained significant. The previously significant interaction effect was no longer significant (*p* = 0.131).

6 Degrees of freedom are for equal variances not assumed (significant Levene’s test).

7 We reanalyzed the data including only those children tested with a 16 s-delay. The main effects of group remained significant. The previously significant interaction effect was no longer significant (*p* = 0.052).

8 Degrees of freedom are for equal variances not assumed.

9 The order in which the conditions were presented did not influence the results when included as a covariate.

## References

[CR1] American Psychiatric Association. (2013). *Diagnostic and Statistical Manual of Mental Disorders* (5th ed.). 10.1176/appi.books.9780890425596.744053

[CR2] Antrop, I., Stock, P., Verté, S., Wiersema, J. R., Baeyens, D., & Roeyers, H. (2006). ADHD and delay aversion: The influence of non-temporal stimulation on choice for delayed rewards. *Journal of Child Psychology and Psychiatry and Allied Disciplines,**47*(11), 1152–1158. 10.1111/j.1469-7610.2006.01619.x 10.1111/j.1469-7610.2006.01619.x 17076754

[CR3] Case, J. P., Laude, J. R., & Zentall, T. R. (2015). Delayed matching to sample in pigeons: Effects of delay of reinforcement and illuminated delays. *Learning and Motivation,**49,* 51–59. 10.1016/j.lmot.2015.01.001

[CR4] Coelho, L. F., Barbosa, D. L. F., Rizzutti, S., Muszkat, M., Amodeo Bueno, O. F., & Miranda, M. C. (2015). Use of cognitive behavioral therapy and token economy to alleviate dysfunctional behavior in children with attention-deficit hyperactivity disorder. *Frontiers in Psychiatry,**6,* 1–9. 10.3389/fpsyt.2015.00167 10.3389/fpsyt.2015.00167 PMC4659172 26635642

[CR5] Cohen, J. (1988). Statistical power analysis for the behavioral sciences (2nd ed.). Lawrence Erlbaum Associates.

[CR6] Cohen, J. (1992). Statistical Power Analysis. *Current Directions in Psychological Science,**1*(3), 98–101. 10.1111/1467-8721.ep10768783

[CR7] Craig, F., Lamanna, A. L., Margari, F., Matera, E., Simone, M., & Margari, L. (2015). Overlap Between Autism Spectrum Disorders and Attention Deficit Hyperactivity Disorder: Searching for Distinctive/Common Clinical Features. *Autism Research,**8*(3), 328–337. 10.1002/aur.1449 10.1002/aur.1449 PMC4654237 25604000

[CR8] De Meyer, H., Beckers, T., Tripp, G., & van der Oord, S. (2019). Deficits in Conditional Discrimination Learning in Children with ADHD are Independent of Delay Aversion and Working Memory. *Journal of Clinical Medicine,**8*(9), 1381. 10.3390/jcm8091381 10.3390/jcm8091381 PMC6780856 31484457

[CR9] Dovis, S., Van Der Oord, S., Wiers, R. W., & Prins, P. J. M. (2012). Can motivation normalize working memory and task persistence in children with attention-deficit/hyperactivity disorder? the effects of money and computer-gaming. *Journal of Abnormal Child Psychology,**40*(5), 669–681. 10.1007/s10802-011-9601-8 10.1007/s10802-011-9601-8 PMC3375007 22187093

[CR10] Esteban, L., Plaza, V., López-Crespo, G., Vivas, A. B., & Estévez, A. F. (2014). Differential outcomes training improves face recognition memory in children and in adults with Down syndrome. *Research in Developmental Disabilities,**35*(6), 1384–1392. 10.1016/j.ridd.2014.03.031 10.1016/j.ridd.2014.03.031 24713518

[CR11] Estévez, A. F., Fuentes, L. J., & Marí-Beffa, P., González, C., & Alvarez, D. (2001). The Differential Outcome Effect as a Useful Tool to Improve Conditional Discrimination Learning in Children. *Learning and Motivation,**32*(1), 48–64. 10.1006/lmot.2000.1060

[CR12] Etkin, M., & D’Amato, M. R. (1969). Delayed matching-to-sample and short-term memory in the capuchin monkey. *Journal of Comparative and Physiological Psychology,**69*(3), 544–549. 10.1037/h0028209

[CR13] Fairbanks, S., & Sugai, G. (2014). Token Economy. *Encyclopedia of Special Education*, 3–4. 10.1002/9781118660584.ese2403

[CR14] Field AP (2013). Discovering statistics using IBM SPSS statistics.

[CR15] Fosco, W. D., Hawk, L. W., Rosch, K. S., & Bubnik, M. G. (2015). Evaluating cognitive and motivational accounts of greater reinforcement effects among children with attention-deficit/hyperactivity disorder. *Behavioral and Brain Functions,**11*(1), 1–9. 10.1186/s12993-015-0065-9 10.1186/s12993-015-0065-9 PMC4438621 25926127

[CR16] Gitten, J. C., Winer, J. L., Festa, E. K., & Heindel, W. C. (2006). Conditional associative learning of spatial and object information in children with attention deficit/hyperactivity disorder. *Child Neuropsychology,**12*(1), 39–56. 10.1080/09297040500205579 10.1080/09297040500205579 16484101

[CR17] Greenhill LL, Nathan PE, Gorman J (1998). Childhood attention deficit hyperactivity disorder: Pharmacological treatments. A guide to treatments that work.

[CR18] Hochhalter, A. K., & Joseph, B. (2001). Differential Outcomes Training Facilitates Memory in People with Korsakoff and Prader-Willi Syndromes. *Integrative Physiological and Behavioral Science,**36*(3), 196–204. 10.1007/BF02734093 10.1007/BF02734093 11777015

[CR19] Holden, J. M., & Overmier, J. B. (2014). Performance under differential outcomes: Contributions of Reward-Specific Expectancies. *Learning and Motivation,**45*(1), 1–14. 10.1016/j.lmot.2013.09.001

[CR20] IBM Corp. (2019). *IBM SPSS Statistics for Macintosh, Version 26.0*. Armonk, NY: IBM Corp.

[CR21] Joseph B, Bruce Overmier J, Thompson T (1997). Food- and nonfood-related differential outcomes in equivalence learning by adults with Prader-Willi syndrome. American Journal on Mental Retardation.

[CR22] Kempton, S., Vance, A., Maruff, P., Luk, E., Costin, J., & Pantelis, C. (1999). Executive function and attention deficit hyperactivity disorder: Stimulant medication and better executive function performance in children. *Psychological Medicine,**29*(3), 527–538. 10.1017/S0033291799008338 10.1017/s0033291799008338 10405075

[CR23] Kort W, Schittekatte M, Bosmans M, Compaan EL, Dekker PH, Vermeir G, Verhaeghe P (2005). WISC-III: handleiding en verantwoording.

[CR24] Luman, M., Oosterlaan, J., & Sergeant, J. A. (2005). The impact of reinforcement contingencies on AD/HD: A review and theoretical appraisal. *Clinical Psychology Review,**25*(2), 183–213. 10.1016/j.cpr.2004.11.001 10.1016/j.cpr.2004.11.001 15642646

[CR25] Luman, M., Tripp, G., & Scheres, A. (2010). Identifying the neurobiology of altered reinforcement sensitivity in ADHD: A review and research agenda. *Neuroscience and Biobehavioral Reviews,**34*(5), 744–754. 10.1016/j.neubiorev.2009.11.021 10.1016/j.neubiorev.2009.11.021 19944715

[CR26] Maki P, Overmier JB, Delos S, Gutmann JA (1995). Expectancies as Factors Influencing Conditional Discrimination Performance of Children. The Psychological Record.

[CR27] Marco, R., Miranda, A., Schlotz, W., Melia, A., Mulligan, A., Müller, U., & Sonuga-Barke, E. J. S. (2009). Delay and reward choice in ADHD: An experimental test of the role of delay aversion. *Neuropsychology,**23*(3), 367–380. 10.1037/a0014914 10.1037/a0014914 19413450

[CR28] Martínez, L., Estévez, A. F., Fuentes, L. J., & Overmier, J. B. (2009). Improving conditional discrimination learning and memory in five-year-old children: Differential outcomes effect using different types of reinforcement. *Quarterly Journal of Experimental Psychology,**62*(8), 1617–1630. 10.1080/17470210802557827 10.1080/17470210802557827 19214832

[CR29] Martínez, L., Flores, P., González-Salinas, C., Fuentes, L. J., & Estévez, A. F. (2013). The effects of differential outcomes and different types of consequential stimuli on 7-year-old children’s discriminative learning and memory. *Learning and Behavior,**41*(3), 298–308. 10.3758/s13420-013-0105-y 10.3758/s13420-013-0105-y 23494477

[CR30] Martínez, L., Marí-Beffa, P., Roldán-Tapia, D., Ramos-Lizana, J., Fuentes, L. J., & Estévez, A. F. (2012). Training with differential outcomes enhances discriminative learning and visuospatial recognition memory in children born prematurely. *Research in Developmental Disabilities,**33*(1), 76–84. 10.1016/j.ridd.2011.08.022 10.1016/j.ridd.2011.08.022 22093651

[CR32] Miller, O. T., Waugh, K. K. M., & Chambers, K. (2002). Differential outcomes effect: Increased accuracy in adults learning kanji with stimulus specific rewards. *The* *Psychological Record,**52*(3), 315–324. 10.1007/BF03395433

[CR33] Mok, L. W., Estevez, A. F., & Overmier, J. B. (2017). Unique Outcome Expectations as a Training and Pedagogical Tool. *The Psychological Record,**60*(2), 227–247. 10.1007/bf03395705

[CR34] Mok LW, Overmier JB (2007). The differential outcomes effect in normal human adults using a concurrent-task within-subjects design and sensory outcomes. Psychological Record.

[CR35] Mok, L. W., Thomas, K. M., Lungu, O. V., & Overmier, J. B. (2009). Neural correlates of cue-unique outcome expectations under differential outcomes training: An fMRI study. *Brain Research,**1265,* 111–127. 10.1016/j.brainres.2008.12.072 10.1016/j.brainres.2008.12.072 19401182

[CR36] Molina, M., Plaza, V., Fuentes, L. J., & Estévez, A. F. (2015). The differential outcomes procedure enhances adherence to treatment: A simulated study with healthy adults. *Frontiers in Psychology*, *6*(NOV), 1–7. 10.3389/fpsyg.2015.01780 10.3389/fpsyg.2015.01780 PMC4753554 26913010

[CR37] Neitzel J (2010). Reinforcement for children and youth with autism spectrum disorders: Online training module.

[CR38] Nigg, J. T., & Casey, B. J. (2005). An integrative theory of attention-deficit/hyperactivity disorder based on the cognitive and affective neurosciences. *Development and Psychopathology,**17*(3), 785–806. 10.1017/S0954579405050376 10.1017/S0954579405050376 16262992

[CR39] Oosterlaan J, Baeyens D, Scheres A, Antrop I, Roeyens H, Sergeant J (2008). VvGK6-16: vragenlijst voor gedragsproblemen bij kinderen 6 tot en met 16 jaar.

[CR40] Overmier, J. B., & Linwick, D. (2001). Conditional Choice-Unique Outcomes Establish Expectancies That Mediate Choice Behavior. *Integrative Physiological and Behavioral Science,**36*(3), 173–181. 10.1007/BF02734091 10.1007/BF02734091 11777013

[CR41] Plaza, V., Molina, M., Fuentes, L. J., & Estévez, A. F. (2018). Learning and recall of medical treatment-related information in older adults using the differential outcomes procedure. *Frontiers in Psychology*, *9*(FEB). 10.3389/fpsyg.2018.00157 10.3389/fpsyg.2018.00157 PMC5817093 29491846

[CR42] Roberts, W. A., & Grant, D. S. (1978). An analysis of light-induced retroactive inhibition in pigeon short-term memory. *Journal of Experimental Psychology: Animal Behavior Processes,**4*(3), 219–236. 10.1037/0097-7403.4.3.219

[CR43] Sagvolden, T., Johansen, E. B., Aase, H., & Russell, V. A. (2005). A dynamic developmental theory of attention-deficit/hyperactivity disorder (ADHD) predominantly hyperactive/impulsive and combined subtypes. *Behavioral and Brain Sciences,**28*(3), 397–419. 10.1017/S0140525X05000075 10.1017/S0140525X05000075 16209748

[CR44] Sattler J (2001). Assessment of Children: cognitive applications.

[CR45] Shaffer, D., Fisher, P., Lucas, C. P., Dulcan, M. K., & Schwab-Stone, M. E. (2000). NIMH Diagnostic Interview Schedule for Children Version IV (NIMH DISC-IV): Description, Differences From Previous Versions, and Reliability of Some Common Diagnoses. *Journal of the American Academy of Child & Adolescent Psychiatry,**39*(1), 28–38. 10.1097/00004583-200001000-00014 10.1097/00004583-200001000-00014 10638065

[CR46] Skinner, B. F. (1950). Are theories of learning necessary? *Psychological Review,**57*(4), 193–216. 10.1037/h0054367 10.1037/h0054367 15440996

[CR47] Slusarek, M., Velling, S., Bunk, D., & Eggers, C. (2001). Motivational effects on inhibitory control in children with ADHD. *Journal of the American Academy of Child and Adolescent Psychiatry,**40*(3), 355–363. 10.1097/00004583-200103000-00016 10.1097/00004583-200103000-00016 11288778

[CR48] Sonuga-Barke, E. J. S. (2002). Psychological heterogeneity in AD/HD—a dual pathway model of behaviour and cognition. *Behavioural Brain Research*, *130*(1–2), 29–36. 10.1016/S0166-4328(01)00432-6 10.1016/s0166-4328(01)00432-6 11864715

[CR49] Staff, A. I., Van den Hoofdakker, B. J., van der Oord, S., Hornstra, R., Hoekstra, P. J., Twisk, J. W. R., Oosterlaan, J., & Luman, M. (2021). Effectiveness of specific techniques in behavioral teacher training for childhood ADHD: A randomized controlled microtrial. *Journal of Clinical Child & Adolescent Psychology,* 1–17. 10.1080/15374416.2020.1846542 10.1080/15374416.2020.1846542 PMC8802898 33471581

[CR50] Sullivan, M. A., & O’Leary, S. G. (1990). Maintenance following reward and cost token programs. *Behavior Therapy,**21*(1), 139–149. 10.1016/S0005-7894(05)80195-9

[CR51] Trapold, M. A. (1970). Are expectancies based upon different positive reinforcing events discriminably different? *Learning and Motivation,**1*(2), 129–140. 10.1016/0023-9690(70)90079-2

[CR52] Urcuioli, P. J. (2005). Behavioral and associative effects of differential outcomes in discrimination learning. *Animal Learning & Behavior,**33*(1), 1–21. 10.3758/bf03196047 10.3758/bf03196047 15971490

[CR53] Vivas, A. B., Ypsilanti, A., Ladas, A. I., Kounti, F., Tsolaki, M., & Estévez, A. F. (2018). Enhancement of Visuospatial Working Memory by the Differential Outcomes Procedure in Mild Cognitive Impairment and Alzheimer’s Disease. *Frontiers in Aging Neuroscience,**10*(November), 1–7. 10.3389/fnagi.2018.00364 10.3389/fnagi.2018.00364 PMC6262409 30524264

[CR54] Wehmeier, P. M., Schacht, A., & Barkley, R. A. (2010). Social and Emotional Impairment in Children and Adolescents with ADHD and the Impact on Quality of Life. *Journal of Adolescent Health,**46*(3), 209–217. 10.1016/j.jadohealth.2009.09.009 10.1016/j.jadohealth.2009.09.009 20159496

[CR55] Willcutt, E. G., Doyle, A. E., Nigg, J. T., Faraone, S. V., & Pennington, B. F. (2005). Validity of the Executive Function Theory of Attention-Deficit/Hyperactivity Disorder: A Meta-Analytic Review. *Biological Psychiatry,**57*(11), 1336–1346. 10.1016/j.biopsych.2005.02.006 10.1016/j.biopsych.2005.02.006 15950006

[CR56] Zar, J. H. (1984). Biostatistical Analysis. *Journal of the American Statistical Association* (2nd ed.). 10.2307/2285423

