# Supplementary material for: Conditional Learning Deficits in Children with ADHD can be Reduced Through Reward Optimization and Response-Specific Reinforcement
Source: Res Child Adolesc Psychopathol. 2021 Apr 1;49(9):1165–78. doi: 10.1007/s10802-021-00781-5 (PMC8322018; doi:10.1007/s10802-021-00781-5)
Supplement: Supplementary file 1 — Supplementary file1 (DOCX 19 KB) [file 10802_2021_781_MOESM1_ESM.docx]

**Appendix**

**Tables**

| Table 1  *Differences in Demographic and Clinical Characteristics between Children in the two Differential Outcomes Conditions* | | | | | | | | | |
| --- | --- | --- | --- | --- | --- | --- | --- | --- | --- |
|  |  | DO – secondary | |  | DO – primary & secondary | |  | *F/χ^2^* | *p* |
| ADHD | N | 24 | |  | 21 | |  |  |  |
|  | Age (M/SD) | 10.29 | 0.95 |  | 10.29 | 1.06 |  | 0.00 | 0.984 |
|  | FSIQ (M/SD) | 98.71 | 12.38 |  | 97.19 | 11.17 |  | 0.18 | 0.670 |
|  | Gender (M:F) | 16:8 | |  | 15:6 | |  | 0.12 | 0.731 |
| TD | N | 26 | |  | 23 | |  |  |  |
|  | Age (M/SD) | 10.06 | 1.10 |  | 10.09 | 1.35 |  | 0.01 | 0.925 |
|  | FSIQ (M/SD) | 105.50 | 11.54 |  | 105.17 | 7.74 |  | 0.01 | 0.909 |
|  | Gender (M:F) | 11:15 | |  | 12:11 | |  | 0.48 | 0.490 |
| Note. ADHD = Attention Deficit Hyperactivity Disorder; TD = Typically Developing; FSIQ: Full Scale IQ; M = Male; F = Female. | | | | | | | | | |

**Instructions**

*Task instruction in the primary and secondary DO condition (translated from Dutch)*

For every yellow smiley you will earn yellow points and for every blue smiley you will earn blue points. At the end, you can exchange all the yellow points for toys and all the blue points for candy. The more yellow points you earn, the higher the chance you have at earning toys. The more blue points you earn, the higher the chance you have at earning candy.

*Task instruction in the secondary DO condition (translated from Dutch)*

For every blue smiley you will earn blue points and for every yellow smiley you will earn yellow points. At the end, you can exchange all the blue points and all the yellow points together for toys and candy. The more points you earn, the higher the chance you have at earning candy and toys

*Task instruction in the nDO condition (translated from Dutch)*

For every smiley you will earn a point. At the end, you can exchange all the points for candy or toys. The more points you earn, the higher the chance you have at candy or toys.
